# Changes in Functionality of Germinated and Non-Germinated Brown Rice Fermented by *Bacillus natto*

**DOI:** 10.3390/foods10112779

**Published:** 2021-11-12

**Authors:** Huei-Ju Wang, Lin Chang, Yu-Shiun Lin

**Affiliations:** Department of Applied Science of Living, Chinese Culture University, 55, Hwa-Kang Road, Yang-Ming-Shan, Taipei 11114, Taiwan; wang31501@gmail.com (L.C.); lyx36@ulive.pccu.edu.tw (Y.-S.L.)

**Keywords:** germinated brown rice, brown rice, *Bacillus natto*, fermentation

## Abstract

Germinated brown rice (GBR) is brown rice (BR) that has been germinated. GBR accumulates more nutrients and has a softer texture than BR. The aim of this study was to ferment GBR and BR using *Bacillus natto* and to investigate the functionality of the fermented products compared with white rice (WR) as a control. After fermentation with *B. natto*, the crude ash, total essential amino acids, and fat contents of each sample increased, while the crude protein content decreased. Moreover, the γ-aminobutyric acid and γ-oryzanol contents decreased, while the 2,2-diphenyl-1-picrylhydrazyl (DPPH) free radical scavenging increased significantly in all fermented samples. The nattokinase activity (FU/g) of the fermented products was highest for GBR (43.11), followed by BR (19.62), and lowest for WR (12.24). Collectively, these results indicate that GBR fermented with *B. natto* yields better nutritional value and functional properties than fermented BR or WR.

## 1. Introduction

With an increase in people’s awareness of health, the preference for whole grains over refined food is increasing in popularity, and brown rice (BR) and germinated brown rice (GBR) have gradually replaced white rice (WR) as staple foods [1]. BR, which has light-brown coloration, has the inedible outer husk removed after harvesting, retaining the bran, aleurone layers, germ, and endosperm. Rice bran is nutrient-rich, containing polyunsaturated fatty acids, a vitamin B complex, vitamin E, γ-oryzanol, dietary fiber, etc. [2]. As a result, BR has many physiological functions, such as adjusting the intestinal flora to stimulate bowel movements [3] and the prevention of cancer [4]. In addition, since the glycemic index of BR (55) is lower than that of refined WR (64), BR delays the rise in blood sugar, which aids blood glucose stabilization in patients with type II diabetes [5]. However, because BR has a rough texture, is unpalatable, and is not easily masticated, it has not been widely adopted. To combat this, researchers have found that germination treatment can improve these sensory shortcomings of BR. GBR is attained by soaking the whole kernel of BR in water until its embryo begins to bud. During germination, the chemical compositions of rice change drastically. Hydrolytic enzymes are activated to decompose large molecular substances into small molecular compounds [6]. The content of the significant and functional component, γ-aminobutyric acid (GABA), is substantially increased with increasing germination time. It has been reported that GABA is an inhibitory transmitter in the mature brain [7].

*Bacillus* species are widely used by the fermentation industry. *Bacillus subtilis* subsp. *natto* (*B. natto*) is commonly used in the commercial production of Japanese food. During the growth process, *B. natto* is capable of producing extracellular enzymes that decompose proteins, carbohydrates, fats, and other macromolecules. Natto, a common fermented soy product with *B. natto*, is rich in amino acids, organic acids, oligosaccharides, and other components that are easily absorbed by the body. In particular, natto contains nattokinase (NK), which was first discovered by Sumi et al. [8]. A large number of studies have confirmed that NK has many beneficial effects on cardiovascular health. NK has potent antithrombotic [9], antihypertensive [10], anti-atherosclerotic, lipid-lowering [11], and neuroprotective [12] activities. Moreover, it has been reported that the ether extracts of rice bran subjected to fermentation with *B. natto* show good antioxidant activity [13]. Rice grains contain fiber and antioxidants, such as ferulic acid, phytic acid, tocopherol, oryzanol, which are known for their anticancer properties [14,15]. Therefore, the fermentation of rice with *B. natto* is of great interest.

The rice samples in this experiment are all of the *Japonica* rice variety. GBR and BR were used as test subjects, WR was used as a control, and the *B. natto* strain was added for fermentation. The nutrient composition (protein, fat, ash, and moisture), functional ingredients (γ-oryzanol, GABA, and free amino acids), and biological activity (DPPH radical scavenging capability and nattokinase activity) of each of the three rice materials were compared before and after fermentation.

## 2. Materials and Methods

### 2.1. Samples

BR (*Oryza sativa*, Tainan 11) was harvested in 2018, and GBR samples were supplied by Asia Rice Biotech Company (Taipei, Taiwan). According to the Asian Rice Biotech Company, the GBR was produced under the condition that the brown rice was completely germinated in water for 22 h at a temperature of 37 °C. WR was obtained after removing the bran of BR by milling for 10 s using a model MR1000E polishing machine (Hosokawa Company, Ltd., Tokyo, Japan). The rice samples were vacuum-packed and stored at −18 °C. Rice powder was obtained as follows: 50 g of rice grain was pulverized with a blade mill (fixed at 20,000 rpm), grinding for 5 s twice.

### 2.2. Chemicals and Standards

Analytical-standard γ-oryzanol was purchased from Wako Pure Chemical Industries (Osaka, Japan). Standards of amino acids and GABA were obtained from Sigma-Aldrich (St. Louis, MO, USA). Certified HPLC-grade solvents n-hexane and isopropanol were purchased from Echo Chemical (Miaoli, Taiwan). All other reagents used for the extraction and analysis were analytical grade or purer and were purchased from Mallinckrodt Pharmaceuticals (Dublin, Ireland) and J. T. Baker Chemicals (Center Valley, PA, USA). Nutrient broth (NB) was purchased form Acumedia Manufacturers (Lansing, MI, USA).

### 2.3. Fermentation

In the present study, the *B. natto* (CKU-20) strain, obtained from Dr. Shih’s lab at the Department of Nutrition and Health Sciences, Chinese Culture University (Taipei, Taiwan), was used as the test organism. To prepare the inoculum, two successive transfers of the test organism were performed in NB at 40 °C and 150 rpm for 24 h. The activated culture was then inoculated into NB and incubated at 40 °C for 16 h, when the viable population was ca. 108 CFU/mL. Rice samples were fermented with *B. natto* following the procedures described by Hung [16]. After rinsing the rice grains with tap water for 30 s, the washed rice samples (100 g) were mixed with 100 mL of medium (0.1% (*w*/*w*) peptone, 0.1% (*w*/*w*) yeast extract) and then sterilized in an autoclave at 121 °C for 20 min. After cooling, the steamed rice samples were inoculated with the test organism by evenly spraying with a 1 mL spore suspension of *B. natto*. After thorough mixing, the inoculated rice substrate was placed into a rotary incubator and then incubated for 60 h at 37 °C, 95% RH, and 150 rpm. The fermented samples were aged in a 4 °C refrigerator for 24 h and then subjected to freeze-drying. The fermentation was conducted in triplicate for each rice sample.

### 2.4. Chemical Composition Determination

The chemical composition was determined according to the AACC method [17]. The moisture content was determined using a hot air oven at 130 °C for 1 h. The protein content (N × 5.95) was analyzed by the Kjeldahl method. The crude fat content was determined with a Soxhlet extraction method. The ash content was measured at 590 °C for 8 h. All analyses were performed in triplicate.

### 2.5. Analysis of Free Amino Acids and GABA

The extraction procedure was modified from Jannoey et al. [18]. Rice powder (250 mg) was placed in 800 μL of 70% (*v/v*) ethanol solution. The mixture was mixed for 1 min and then centrifuged at 13,000× *g* at 4 °C for 10 min. The supernatant was collected. The above extraction was repeated. The collected supernatant (3 mL) was filtered and analyzed by liquid chromatography–electrospray ionization tandem mass spectrometry (LC-ESI-MS).

For the identification of free amino acids and GABA, a Finnigan LXQ linear-ion trap mass spectrometer (Thermo Scientific, Waltham, MA, USA) was employed. The operation conditions of the MS detector were fragmentation range: 70, mass range: 50–300 *m*/*z*, sheath gas flow rate: 30 arb unit, auxiliary gas flow rate: 12 arb unit, sweep gas flow rate: 1 arb unit, spray voltage: 5.5 kV, capillary voltage: −17 V, and capillary temperature: 290 °C.

Free amino acids and GABA were analyzed with the Survey HPLC system and an Aquasil C18 column (250 × 2.1 mm, 5 μm) (Thermo Fisher Scientific, Waltham, MA, USA). A linear gradient system was used with mobile phase A (0.1% ammonium perfluorovalerate in water) and mobile phase B (0.1% ammonium perfluorovalerate in acetonitrile); A: 95–85%, 85–40%, and 40–95% were used at 0–10 min, 10–20 min, and 20–21 min, respectively, and then 95% A was used for another 9 min. The flow rate was 0.2 mL/min and the sample volume was 5 μL. The data were acquired and processed by using Xcalibur software (Thermo Fisher Scientific, Waltham, MA, USA).

### 2.6. Analysis of γ-Oryzanol

Extraction of γ-oryzanol was performed according to AACC Method 30-10 [17] and the method of Heinemann et al. [19] with a slight modification. The rice sample (4 g) was added with 8 mL of 95% ethyl alcohol and stirred. Next, 20 mL of 4 N HCl was added and heated in a water bath at 70–80 °C for 90 min. After cooling, the mixture was added to 20 mL of 95% ethyl alcohol. Subsequently, petroleum ether (25 mL) was added and shaken vigorously for 1 min. The supernatant was centrifuged at 10,000× *g* for 20 min. The above steps were repeated twice. The supernatants were combined and evaporated to dryness. The dried material was dissolved in 2 mL of HPLC-grade hexane. An aliquot of the sample was filtered for HPLC analysis.

γ-Oryzanol was analyzed using a silica-gel column (250 × 4.6 mm, 5 μm, SPS100-5, Chromatorex, Thermo Fisher Scientific, Waltham, MA, USA) at 30 °C and a KNAUER Smartline series 1000 HPLC (Advanced Scientific, Berlin, Germany) equipped with a photodiode array detector (Jasco MD-215 plus, Tokyo, Japan). Detection was accomplished by measuring the absorbance at 330 nm. The mobile phase was ethyl acetate/acetic acid/n-hexane (isocratic at 1.8:1.8:98.4 (*v*/*v*/*v*)), with a flow rate of 1.5 mL/min. The content was quantified by comparison of the peak area with that of a γ-oryzanol standard curve. The standard curve concentration of γ-oryzanol was formulated in five standard solutions with concentrations ranging from 0–800 ppm.

### 2.7. Determination of DPPH Scavenging Activity

The DPPH scavenging activity of the rice samples was measured as described by Liyana-Pathirana and Shahidi [20] with some modifications. The crude rice extract was prepared by soaking 0.1 g of the sample powder in 1 mL of 50% methanol solution for 1 h and then centrifuging at 7500× *g* for 10 min. The supernatant (0.1 mL) was mixed with 1.9 mL of DPPH (Fluka Chemie, Buchs, Switzerland) in methanol (0.02 mg/mL). The mixtures were left for 30 min and then measured at 517 nm (UV-2250PC, Shimadzu Co., Kyoto, Japan). The scavenging activity was calculated as follows:

% Inhibition = [(blank absorbance − sample absorbance)/blank absorbance] × 100

### 2.8. Determination of Nattokinase Activity

The nattokinase activity was determined according to Jorge et al. [21]. Rice powder (1 g) was suspended in 20 mL of boric acid saline buffer (0.05 M H_3_BO_3_, 0.05 M KCl, pH 7.8), settled for 20 min, and then filtered. Boric acid saline buffer (1.4 mL) and fibrinogen solution (0.05% (*w*/*v*), 0.4 mL) were combined in a vial and kept in a water bath (37 °C) for 5 min. Then, 0.1 mL thrombin (20 U/mL) was added and kept in the water bath (37 °C) for a further 10 min. To this clot, 0.1 mL of sample extract was added and boric acid buffer was as a control. After incubation (37 °C, 60 min), trichloroacetic acid (0.2 M, 2 mL) was added. The vials were kept in the water bath for a further 20 min and then centrifuged at 3000× *g* for 5 min. One unit of enzyme activity is defined as the amount of enzyme required to produce an increase of 1.0 in the absorbance at 275 nm in 60 min.

### 2.9. Statistical Analysis

Data were analyzed using the SAS software version 9 (SAS Institute, Cary, NC, USA). Analysis of variance (ANOVA) and Duncan’s tests were performed. In all cases, the significance was established at *p* ≤ 0.05. All experiments were carried out in triplicate unless otherwise stated.

## 3. Results and Discussion

### 3.1. Chemical Composition

Chemical composition analyses of the rice samples are shown in Table 1. For the unfermented, GBR had the highest content of crude ash, crude fat, and crude protein. Comparable contents of these components were found in BR, whereas the lowest content of each component was observed in WR. It was consistent with the results of Ohtsubo et al. [22]. Nutrients are lost with the removal of bran during the milling process used to obtain WR, and therefore, whole grain rice (both GBR and BR) is recommended to replace refined WR in the general diet [23]. Fermentation caused increases in the crude ash and crude fat content in rice, but a decrease of the crude protein content (Table 1), which is consistent with the results of previous studies [13]. It is likely due to the proteolytic enzyme secreted by *B. natto* during fermentation [24]. As well, *B. natto* fermentation significantly increased the contents of soluble dietary fiber [13].

### 3.2. Content of Free Amino Acids and GABA

The free amino acid contents of rice samples before and after fermentation were measured, as shown in Table 2. For the unfermented, WR had a lower total amount of free amino acids than GBR and BR (*p* < 0.05). In terms of individual free amino acids, compared with BR, GBR had a significantly lower content of glutamic acid, but relatively higher contents of other free amino acids, especially valine, histidine, leucine/isoleucine, and lysine. According to previous reports [25,26], the amino acids in BR mainly exist in the form of proteins. During the germination process, the proteins are broken down, resulting in an increase of free amino acids. Additionally, glutamate decarboxylase is activated, which catalyzes the conversion of glutamic acid into GABA; therefore, GBR has a lower glutamic acid content and a higher GABA content than BR [27].

After fermentation, the total free amino acids (mg/100 g dm) increased in GBR (9.36→15.09) and WR (1.29→5.67) but decreased in BR (11.19→7.23). The softened whole seed coat of GBR and the softer tissues of WR after bran removal compared with that of BR is likely responsible for the observed trend. The total essential amino acid (EAA) contents (mg/100 g dm) of GBR, BR, and WR were increased by ~2.7-fold (4.77→13.00), ~1.3-fold (3.61→4.73), and ~7.5-fold (0.57→4.29), respectively. Therefore, the essential amino acid content is increased in fermented rice which is consistent with the results of previous studies [24,28].

As rice is deficient in lysine, other foods, such as beans, meat, and dairy products, should be used as supplements. Alternatively, fermentation by naturally occurring lactic acid bacteria can significantly increase the lysine content in cereal products, including rice, oats, corn, and wheat [27]. The results in Table 2 show that the free lysine contents in the unfermented samples decreased in the following order: GBR > BR > WR, which is consistent with the results of previous studies, and suggests that the nutrient content of BR increases after germination [24]. In addition, the lysine contents of the fermented rice samples were higher than those of the unfermented samples, with significant differences observed for GBR and WR. This result could be useful for the future development and application of related rice products with enhanced nutritional value.

Table 3 presents the GABA contents of the rice samples (mg/100 g dm). In the unfermented samples, GBR had the highest GABA content (7.21), followed by BR (0.70), and the lowest content (only 0.15) was found in WR. These results are consistent with previous findings, in which the GABA content of GBR is 4–20 times higher than that of BR [25,29].

In this study, we attempted to produce GABA-containing foods from different types of rice using *B. natto* fermentation. The results in Table 3 reveal that the GABA contents of the fermented rice samples were lower than those of the unfermented samples. The GABA contents after fermentation were only about 10%, 29%, and 55% for GBR, BR, and WR, respectively. *B. natto* can produce unique viscous substances with γ-polyglutamic acid (γ-PGA) as the main component [1], and the decrease in GABA content after fermentation may be due to the generation of such substances. Appropriate microorganisms and optimum fermentation conditions need to be chosen for the production of BR-based GABA-rich foods.

### 3.3. γ-Oryzanol Content

γ-Oryzanol is a mixture of phytosteryl ferulates located in rice bran. The γ-oryzanol contents of GBR, BR, and WR before and after fermentation are shown in Table 4. In the unfermented samples, the γ-oryzanol contents (mg/100 g dm) of GBR, BR, and WR were 50.97, 50.80, and 9.13, respectively, with no significant difference found between the γ-oryzanol contents of BR and GBR. γ-Oryzanol is the active substance in rice and mainly exists in rice bran. Ohtsubo et al. [22] soaked Koshihikari rice in water at 30 °C for 72 h; after germination, the γ-oryzanol contents of GBR and BR were measured, and no significant difference was found, which was consistent with the results in this study.

After fermentation, the γ-oryzanol contents of BR, GBR, and WR were reduced to 28.60, 24.66, and 2.65 mg/100 g dm, respectively. The amount of γ-oryzanol in the rice would vary due to the fermentation of different microorganisms. Ilowefah et al. [30] found that the yeast fermentation caused the reduction of the contents of γ-oryzanol of brown rice flour. Furthermore, Sirilun et al. [31] even produced ferulic acid from oryzanol degradation during the fermentation of black rice bran by ferulic acid esterase producing *Aspergillus oryzae* HP. However, Cáceres et al. [32] reported that LAB strains fermentation did not modify notably levels of γ-oryzanol. Massarolo et al. [33] showed an increase of γ-oryzanol content in rice bran fermented with *Rhizopus oryzae*.

### 3.4. DPPH Radical Scavenging Capacity

As shown in Table 5, in the unfermented samples, BR exhibited the highest DPPH scavenging rate, followed by GBR, and the lowest DPPH scavenging rate was obtained for WR. The DPPH scavenging capacities of the fermented GBR, BR, and WR were significantly enhanced compared with those of the corresponding unfermented samples (*p* < 0.05). Many studies have shown that fermentation enhances DPPH radical scavenging capability of rice samples, such as *Lentinula edodes* [34], *Bacillus amyloliquefaciens* [35], *Bacillus subtilis* [36], and *Bacillus natto* [13]. Qi et al. [37] found that allyl methyl sulfide was the main antioxidant component of ethyl acetate phase extracted from fermented rice bran by *Bacillus natto*.

### 3.5. Nattokinase Activity

Nattokinase is only produced during the *B. natto* fermentation process, and nattokinase activity is affected by the fermentation processing conditions and species selection. Therefore, in this study, when the unfermented samples were extracted, the measured absorbance values were negative, and the nattokinase content could not be measured.

As shown in Table 6, GBR exhibited the highest nattokinase activity (43.11 FU/g), followed by BR (19.62 FU/g), and then WR (12.24 FU/g). Wang et al. [38] used fermented peanuts, soybeans, and black beans with *B. natto*, and the obtained nattokinase activities of the three samples were highest in soybeans (42.38 FU/g). The result in this study has shown that *B. natto* fermented GBR has considerable nattokinase activity. They concluded that the nattokinase activity is proportional to the protein content. The correlation between the nattokinase activity of the rice samples in this study and the protein content was analyzed, and a correlation coefficient of 0.70 was obtained, which is consistent with the experimental results of Wang et al. [38]. In addition, a commercially available natto product was analyzed, and its nattokinase activity of 249.31 FU/g was considerably higher than those of the three rice samples (*p* < 0.05). Although the nattokinase activities of the fermented rice samples were not as high as that of the commercial natto, the nattokinase content can be further improved by optimizing the fermentation conditions.

## 4. Conclusions

The nutritional and sensorial properties of cereals and pseudocereals could be enhanced by their germination and fermentation. Thus leads to improved product properties by changing increased nutritional value and better digestibility of the grains making them better food material than the raw grains [39]. In addition, compared with the fermented white rice, the fermented brown rice had higher nutritional components, flavor, and antioxidant activity [25]. In this study, GBR fermented with *B. natto* yields improved nutritional value and better functional properties than fermented BR or WR. Thus, GBR could be used for developing rice-based products with enhanced nutritional value. The *B. natto* fermentation conditions should be further optimized to improve the content of functional components, as although the nattokinase activity of fermented GBR is considerably higher than those of fermented BR and WR, it is lower than that of commercial natto products. In future research, the scope of the study can be expanded to explore the optimum growth conditions for *B. natto* fermentation of GBR, with the potential of promoting different active and/or functional components. Such improvements may then encourage increased production of processed BR products.

## Figures and Tables

**Table 1:** Chemical composition of germinated brown rice (GBR), brown rice (BR), and white rice (WR) before and after fermentation. The results expressed on a dry matter (dm) basis.

Parameter (%)	GBR		BR		WR	
	Unfermented	Fermented	Unfermented	Fermented	Unfermented	Fermented
Moisture	15.00 ± 0.11 ^b^	1.99 ± 0.14 ^C^*	15.33 ± 0.06 ^b^	2.56 ± 0.09 ^B^*	15.75 ± 0.27 ^a^	5.60 ± 0.23 ^A^*
Crude ash	1.27 ± 0.04 ^a^	1.64 ± 0.03 ^A^*	1.23 ± 0.10 ^a^	1.33 ± 0.02 ^B^	0.49 ± 0.04 ^b^	0.46 ± 0.10 ^C^
Crude lipid	1.19 ± 0.24 ^a^	1.39 ± 0.20 ^A^*	0.97 ± 0.10 ^a^	1.15 ± 0.10 ^AB^	0.22 ± 0.06 ^b^	0.89 ± 0.34 ^B^*
Crude protein	8.59 ± 0.50 ^a^	7.59 ± 0.22 ^A^*	8.08± 0.46	7.58 ± 0.18 ^A^	8.20 ± 0.70 ^a^	6.27 ± 0.10 ^B^*

1. Data are expressed as mean ± SD (*n* = 3). 2. For each of the unfermented (a,b) and fermented (A–C) groups, means within a row with the same superscript letter are not significantly different (*p* < 0.05). 3. (*) indicates within each rice sample, the means of the unfermented and fermented groups are significantly different (*p* < 0.05).

**Table 2:** Free amino acid contents (mg/100 g dm) in germinated brown rice (GBR), brown rice (BR), and white rice (WR) before and after fermentation.

Amino Acid	GBR		BR		WR	
	Unfermented	Fermented	Unfermented	Fermented	Unfermented	Fermented
serine	1.32 ± 0.07	0.17 ± 0.00 *	1.03 ± 0.11	0.07 ± 0.01 *	0.16 ± 0.01	0.15 ± 0.01
threonine	0.37 ± 0.07	0.46 ± 0.03	0.40 ± 0.05	0.31 ± 0.01	0.11 ± 0.02	0.23 ± 0.02 *
glutamic acid	1.51 ± 0.08	1.07 ± 0.07	4.74 ± 0.30	1.94 ± 0.02 *	0.38 ± 0.02	0.78 ± 0.04 *
proline	1.05 ± 0.09	0.44 ± 0.02 *	1.06 ± 0.16	0.21 ± 0.01 *	0.12 ± 0.01	0.16 ± 0.00 *
valine	0.91 ± 0.07	2.37 ± 0.04 *	0.49 ± 0.01	0.62 ± 0.00 *	0.06 ± 0.00	0.74 ± 0.07 *
methionine	0.22 ± 0.06	0.80 ± 0.01 *	0.15 ± 0.01	0.26 ± 0.00 *	0.01 ± 0.00	0.30 ± 0.04 *
tyrosine	1.30 ± 0.10	3.05 ± 0.13 *	1.50 ± 0.02	1.42 ± 0.01 *	0.18 ± 0.00	0.92 ± 0.02 *
histidine	0.78 ± 0.01	0.57 ± 0.04 *	0.25 ± 0.18	0.23 ± 0.02	0.08 ± 0.00	0.19 ± 0.03 *
leucine/isoleucine	0.41 ± 0.07	2.04 ± 0.09 *	0.27 ± 0.01	0.65 ± 0.00 *	0.05 ± 0.01	0.84 ± 0.00 *
lysine	0.28 ± 0.01	0.45 ± 0.05 *	0.11 ± 0.08	0.23 ± 0.01	0.02 ± 0.01	0.18 ± 0.01 *
arginine	0.71 ± 0.00	0.41 ± 0.06	0.75 ± 0.03	0.28 ± 0.00	0.06 ± 0.01	0.29 ± 0.02 *
phenylalanine	0.50 ± 0.02	3.26 ± 0.14 *	0.44 ± 0.17	1.01 ± 0.02	0.06 ± 0.00	0.89 ± 0.05 *
Total Free AA	9.36 ± 0.73 ^a^	15.09 ± 0.39 ^A^*	11.19± 0.10 ^a^	7.23 ± 0.00 ^B^*	1.29 ± 0.04 ^b^	5.67 ± 0.13 ^C^*
total EAA	4.77 ± 0.20 ^a^	13.00 ± 0.24 ^A^*	3.61 ± 0.11 ^b^	4.73 ± 0.00 ^B^*	0.57 ± 0.02 ^c^	4.29 ± 0.21 ^B^*

1. Data are expressed as mean ± SD (*n* = 2). 2. For each of the unfermented (a–c) and fermented (A–C) groups, means within a row with the same superscript letter are not significantly different (*p* < 0.05). 3. (*) indicates within each rice sample, the means of the unfermented and fermented groups are significantly different (*p* < 0.05).

**Table 3:** Effect of fermentation on the γ-aminobutyric acid (GABA) contents of rice samples.

Sample	GABA (mg/100 g dm)	
	Unfermented	Fermented
GBR	7.21 ± 0.41 ^a^	0.73 ± 0.07 ^a^*
BR	0.70 ± 0.03 ^b^	0.20 ± 0.01 ^b^*
WR	0.15 ± 0.01 ^b^	0.09 ± 0.00 ^b^*

1. Data are expressed as mean ± SD (*n* = 2). 2. Means within a column with the same superscript letter are not significantly different (*p* < 0.05). 3. (*) indicates means within a row are significantly different (*p* < 0.05). 4. GBR: germinated brown rice, BR: brown rice, WR: white rice.

**Table 4:** Effect of fermentation on the γ-oryzanol contents of rice samples.

Sample	γ-Oryzanol (mg/100 g dm) ^a^	
	Unfermented	Fermented
GBR	50.97 ± 4.89 ^a^	24.66 ± 2.37 ^a^*
BR	50.80 ± 2.11 ^b^	28.60 ± 1.84 ^b^*
WR	9.13 ± 0.41 ^b^	2.65 ± 0.52 ^b^*

1. Data are expressed as mean ± SD (*n* = 3). 2. Means within a column with the same superscript letter are not significantly different (*p* < 0.05). 3. (*) indicates means within a row are significantly different (*p* < 0.05). 4. GBR: germinated brown rice, BR: brown rice, WR: white rice.

**Table 5:** Effect of fermentation on the DPPH-scavenging (%) of rice samples.

Sample	DPPH Scavenging (%) ^a^	
	Unfermented	Fermented
GBR	30.80 ± 0.76 ^b^	83.24 ± 4.26 *
BR	36.95 ± 1.19 ^a^	83.94 ± 2.89 *
WR	11.31 ± 0.46 ^c^	86.04 ± 5.99 *

1. Data are expressed as mean ± SD (*n* = 3). 2. Means within a column with the same superscript letter are not significantly different (*p* < 0.05). 3. (*) indicates means within a row are significantly different (*p* < 0.05). 4. GBR: germinated brown rice, BR: brown rice, WR: white rice.

**Table 6:** Effect of fermentation on the nattokinase activity (FU/g) of rice samples.

Sample	Nattokinase Activity (FU/g)	
	Unfermented	Fermented
GBR	nd	43.11 ± 0.32 ^b^
BR	nd	19.62 ± 0.21 ^c^
WR	nd	2.65 ± 0.30 ^c^
Commercial natto	-	249.31 ± 1.12 ^a^

1. Data are expressed as mean ± SD (*n* = 3). 2. Means within a column with the same superscript letter are not significantly different (*p* < 0.05). 3. GBR: germinated brown rice, BR: brown rice, WR: white rice, nd: not detected.

## References

[1] Shih I.L., Van Y.T. (2001). The production of poly-(γ-glutamic acid) from microorganisms and its various applications. Bioresour. Technol..

[2] Nagendra Prasad M.N., Sanjay K.R., Shravya Khatokar M., Vismaya M.N., Nanjunda Swamy S. (2011). Health benefits of rice bran—A Review. J. Nutr. Food Sci..

[3] Nemoto H., Ikata K., Arimochi H., Iwasaki T., Ohnishi Y., Kuwahara T., Kataoka K. (2011). Effects of fermented brown rice on the intestinal environments in healthy adult. J. Med. Investig..

[4] Lin P.Y., Li S.C., Lin H.P., Shih C.K. (2019). Germinated brown rice combined with *Lactobacillus acidophilus* and *Bifidobacterium animalis* subsp. lactis inhibits colorectal carcinogenesis in rats. Food Sci. Nutr..

[5] Schulze M.B., Liu S., Rimm E.B., Manson J.E., Willett W.C., Hu F.B. (2004). Glycemic index, glycemic load, and dietary fiber intake and incidence of type 2 diabetes in younger and middle-aged women. Am. J. Clin. Nutr..

[6] Smith G., Nkhata E.A., Kamau E.H., Shingiro J.B. (2018). Fermentation and germination improve nutritional value of cereals and legumes through activation of endogenous enzymes. Food Sci. Nutr..

[7] Sears S.M.S., Hewett S.J. (2021). Influence of glutamate and GABA transport on brain excitatory/inhibitory balance. Exp. Biol. Med..

[8] Sumi H., Hamada H., Tsushima H., Mihara H., Muraki H. (1987). A novel fibrinolytic enzyme (nattokinase) in the vegetable cheese Natto; a typical and popular soybean food in the Japanese diet. Experientia.

[9] Chen H., McGowan E.M., Ren N., Lal S., Nassif N., Shad-Kaneez F., Qu X., Lin Y. (2018). Nattokinase: A promising alternative in prevention and treatment of cardiovascular diseases. Biomark Insights.

[10] Shahbazi R., Sharifzad F., Bagheri R., Alsadi N., Yasavoli-Sharahi H., Matar C. (2021). Anti-Inflammatory and Immunomodulatory Properties of Fermented Plant Foods. Nutrients.

[11] Ren N., Chen H., Li Y., McGowan E., Lin Y. (2017). A clinical study on the effect of nattokinase on carotid artery atherosclerosis and hyperlipidaemia. Chin. Med. J..

[12] Fadl N., Ahmed H., Booles H., Sayed A. (2013). Serrapeptase and nattokinase intervention for relieving Alzheimer’s disease pathophysiology in rat model. Hum. Exp. Toxicol..

[13] Chu J., Zhao H., Lu Z., Lu F., Bie X., Zhang C. (2019). Improved physicochemical and functional properties of dietary fiber from millet bran fermented by *Bacillus natto*. Food Chem..

[14] Katayama M., Yoshimi N., Yamada Y., Sakata K., Kuno T., Yoshida K., Qiao Z., Vihn P.Q., Iwasaki T., Kobayashi H. (2002). Preventive effect of fermented brown rice and rice bran against colon carcinogenesis in male F344 rats. Oncol. Rep..

[15] Chung S.I., Lee S.C., Yi S.J., Kang M.Y. (2018). Antioxidative and antiproliferative activities of ethanol extracts from pigmented giant embryo rice (*Oryza sativa* L. cv. Keunnunjami) before and after germination. Nutr. Res. Pract..

[16] Hung C.L. (2006). Microbiological Control in the Production Process of Germinated Brown Rice and the Study on its Bioactive Fermented Products. Ph.D. Thesis.

[17] AACC (2003). Approved Methods of the American Association of Cereal Chemistry.

[18] Jannoey P., Niamsup H., Lumyong S., Suzuki T., Katayama T., Chairote G. (2010). Comparison of gamma-aminobutyric acid production in Thai rice grains. World J. Microbiol. Biotechnol..

[19] Heinemann R.J.B., Xu Z., Godber J.S., Lanfer-Marquez U.M. (2008). Tocopherols, tocotrienols and γ-oryzanol contents in Japonica and Indica subspecies of rice (*Oryza sativa* L.) cultivated in Brazil. Cereal Chem..

[20] Liyana-Pathirana C.M., Shahidi F. (2007). Antioxidant and free radical scavenging activities of whole wheat and milling fractions. Food Chem..

[21] Jorge G.H., Chandra J.H., Heinz E., Mien M., Suwandi H., Govind R. (1993). Isolation and characterization of hydroxymethylglutary-coenzyme-A reductase inhibitors from fermented soy bean extracts. J. Clin. Biochem. Nutr..

[22] Ohtsubo K., Suzuki K., Yasui Y., Kasumi T. (2005). Bio-functional components in the processed pre-germinated brown rice by a twin-screw extruder. J. Food Compo. Anal..

[23] Upadhyay A., Karn S.K. (2018). Brown Rice: Nutritional composition and Health Benefits. J. Food Sci. Technol..

[24] Xu Y., Jin Y., Su J., Yang N., Xu X., Jin Z., Cui B., Wu F. (2021). Changes in the nutritional value, flavor, and antioxidant activity of brown glutinous rice during fermentation. Food Biosci..

[25] Komatsuzaki N., Tsukahara K., Toyoshima H., Suzuki T., Shimizu N., Kimura T. (2007). Effect of soaking and gaseous treatment on GABA content in germinated brown rice. J. Food Eng..

[26] Sharma S., Jan R., Riar C., Bansal V. (2021). Analyzing the effect of germination on the pasting, rheological, morphological and in- vitro antioxidant characteristics of kodo millet flour and extracts. Food Chem..

[27] Roohinejad S., Omidizadeh A., Mirhosseini H., Saari N., Mustafa S., Hussin A.S.M., Hamid A., Manap M.Y.A. (2011). Effect of pre-germination time on amino acid profile and gamma amino butyric acid (GABA) contents in different varieties of Malaysian brown rice. Int. J. Food Prop..

[28] Hamad A.M., Fields M.L. (1979). Evaluation of the protein quality and available lysine of germinated and fermented cereals. J. Food Sci..

[29] Saikusa T., Horino T., Mori Y. (1994). Accumultion of γ-aminobutyric acid (GABA) in the rice germ during water soaking. Biosci. Biotechnol. Biochem..

[30] Ilowefah M., Bakar J., Ghazali H.M., Mediani A., Muhammad K. (2015). Physicochemical and functional properties of yeast fermented brown rice flour. J. Food Sci. Technol..

[31] Sirilun S., Chaiyasut C., Pengkumsri N., Pelyuntha W., Peerajan S., Sivamaruthi B.S. (2015). Production of ferulic acid from oryzanol degradation during the fermentation of black rice bran by ferulic acid esterase producing *Aspergillus oryzae* HP. J. Pure Appl. Microbiol..

[32] Cáceres P.J., Peñas E., Martínez-Villaluenga C., García-Mora P., Frías J. (2019). Development of a multifunctional yogurt-like product from germinated brown rice. LWT.

[33] Massarolo K.C., de Souza T.D., Collazzo C.C., Furlong E.B., de Souza Soares L.A. (2017). The impact of *Rhizopus oryzae* cultivation on rice bran: Gamma-Oryzanol recovery and its antioxidant properties. Food Chem..

[34] Jung T.-D., Shin G.-H., Kim J.-M., Choi S.-I., Lee J.-H., Lee S.J., Park S.J., Woo K.S., Oh S.K., Lee O.-H. (2017). Comparative analysis of γ-oryzanol, β-glucan, total phenolic content and antioxidant activity in fermented rice bran of different varieties. Nutrients.

[35] Wu S., Lu S., Liu J., Yang S., Yan Q., Jiang Z. (2021). Physicochemical properties and bioactivities of rice beans fermented by *Bacillus amyloliquefaciens*. Engineering.

[36] Gum S.I., Nguyen P.A., Lee J.R., Han Y.H., Cho M.K. (2017). The physico-chemical alteration of lovastatin and enhanced antioxidant effect of Bacillus subtilis fermented-red yeast rice product. Food Chem..

[37] Qi H.B., Song J.X., Chen J. (2012). Study on the antioxidant component of fermented rice bran. Chin. Food Sci..

[38] Wang C.Y., Chuang P.T., Chan C.H., Tseng C.Y. (2005). Studies on the fermentation of peanut (*Arachis hypogaea*) by *Bacillus subtilis natto*. Taiwan J. Agric. Chem. Food Sci..

[39] Singh A.k., Rehal J., Kaur A., Jyot G. (2015). Enhancement of attributes of cereals by germination and fermentation: A review. Crit. Rev. Food Sci. Nutr..

